# Equal Contributions and Credit: An Emerging Trend in the Characterization of Authorship in Major Anaesthesia Journals during a 10-Yr Period

**DOI:** 10.1371/journal.pone.0071430

**Published:** 2013-08-05

**Authors:** Zhi Li, Yu-Ming Sun, Fei-Xiang Wu, Li-Qun Yang, Zhi-Jie Lu, Wei-Feng Yu

**Affiliations:** Department of Anaesthesiology, Eastern Hepatobiliary Surgery Hospital, Second Military Medical University, Shanghai, China; University Medical Center Göttingen, Germany

## Abstract

**Background:**

The practice of giving certain authors equal credit in original research publications was increasingly common in some specialty. This study aimed to investigate the prevalence and characteristics of designating some authors with equally credited authors (ECAs) in major anaesthesia journals.

**Methodology/Principal Findings:**

The practice of giving authors equal credit was searched and identified in the three major anaesthesia journals between January 1, 2002 and December 31, 2011. Papers with ECAs had a higher proportion of the total number of articles in 2011 versus published in 2002 (*Anesthesiology*, 8.8% vs. 0.9%; *British Journal of Anaesthesia*, 8.8% vs. 0%; *Anesthesia & Analgesia*, 3.4% vs. 0.3%; totally, 6.4% vs. 0.4%). A significant increasing trend in annual proportion of articles with ECA was found in the three journals. The first two authors listed in the byline had equal credit in most cases.

**Conclusions/Significance:**

The practice of giving authors equal credit in original research papers is increasingly common in major anaesthesia journals. It may be warranted for the journals to guide the authors how to regard this practice.

## Introduction

The outside perception of individual academic contribution is undisputedly a critical consideration for researchers, especially in evaluating academic promotion. The detailed contribution to the conception, design, analysis and interpretation is called to be listed on the byline of an original paper or in the cover letter [1]. The credit of authors' contributions to an original article has been a matter of great dispute [2], [3]. Generally, it is widely considered that the first author and last author in an article's byline are the most important with special weight. The other authors were usually ranked according to their contribution to the manuscript.

Sometimes, it is difficult to order the authors in a manuscript, especially when two or more authors were of similar contribution that they could be considered as co-first authors. Thus, it is not surprising to see some articles with two or more authors who “contributed equally” to the work. A recent report showed that the practice of giving certain authors equal credit in original research publications was increasingly common in the five top general medicine journals [4]. Another study showed that it is also increasingly popular to give authors equal credit in the major four critical care medicine journals [5]. However, whether this trend also existed in the major journals of anesthesiology was unknown yet. Hence, we performed this study aimed to investigate the prevalence and characteristics of the practice designating equally credited authors (ECAs) in the field of anesthesiology.

## Methods

We focused specifically on the major anaesthesia journals with current highest average impact factors. These journals were *British Journal of Anaesthesia* (*BJA*), *Anesthesiology* (*Ane*), *Anesthesia & Analgesia* (*A&A*) and *Anaesthesia* (*Ana*). In the journal *Anaesthesia*, statements of ECAs are forbidden [6]. Therefore, we restricted the literature search in the remaining three journals. All the original research papers published from Jan 1, 2002 to Dec 31, 2011 were screened on the three journals' Websites by reading the author information, acknowledgment, and footnote, to search the articles with ECA authors. All articles identified by the online search were further evaluated to ensure that they met the inclusion criteria: 1) they were original papers, including systematic reviews but not clinical reviews or case reports; and 2) statements designating some authors for equal credit were used in the correct context. The structure of this paper was remodeled on two previous publications [4], [5].

For each journal, the yearly number of articles which met the inclusion criteria was calculated from 2002 to 2011. For each of these articles, we also extracted the following information: total number of authors, the number of authors with ECA and their positions in the byline, affiliation of the corresponding author, and year of publication.

The proportion of articles with ECAs was calculated in each journal per year. We used the online literature database (ISI Web of Science) to gain the yearly total number of original articles in each journal. We restricted the search under the headings “articles” and “reviews”. Besides, we also screened the region origins of articles with ECAs. Finally, we read the three journals' instructions to authors, investigating if they demanded authors to state their individual contributions and whether they supplied any guide to regard the practice of giving some authors equal credit.

### Statistical analysis

Because our only goal is to describe trends and not to test hypotheses about the relative contribution of different countries, simple descriptive statistics (eg, sum or average) are mainly used. The Cochran-Armitage trend test for the trends of the proportion of articles with ECA for each journal from 2002 to 2011 was applied [5].

## Results

Original articles with authors given equal credit were found in three journals, except *Anaesthesia*. The most regular statements used were that several authors contributed equally to the study (manuscript, work, or article) or had equal contribution to the study. The other statements were rather few, including “co-first authors” and “co-senior authors”.

The three anaesthesia journals published 10,945 original research articles over the study period. In total, there were 232 original research articles with ECAs (106 in *Ane*, 59 in *BJA* and 67 in *A&A*). Among these articles, the ‘equally contributing’ authors in 86 articles were from different institutions (39 in *Ane*, 21 in *BJA* and 26 in *A&A*).

**Figure 1:**
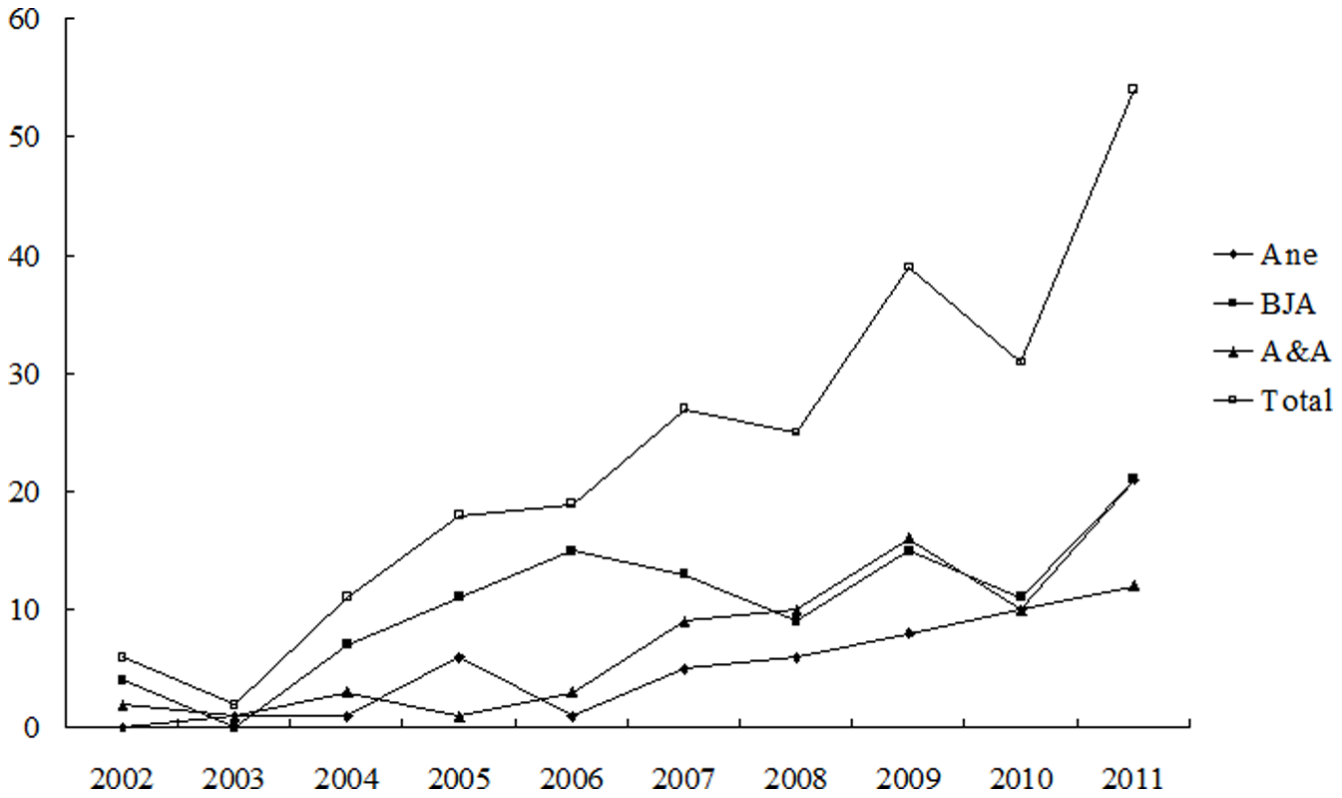
Yearly number of original research papers with ECAs published in major anaesthesia journals in 2002–2011.

For all the three anaesthesia journals, there were significant increases in original research papers with ECAs in the proportion of the overall number of articles published in 2011 versus in 2002: *Ane*, 8.8% vs. 0.9%; *BJA*, 8.8% vs. 0%; *A&A*, 3.4% vs. 0.3%; totally, 6.4% vs. 0.4%. (Table 1, Figure 1) There were also statistically significant increasing trends in annual proportion of ECA papers for all the three journals.

**Table 1:** Number of original research articles with authors given equal credit and annual prevalence.

Year	*Ane*	*BJA*	*A&A*	Total
2002	4/429(0.9%)	0/261(0)	2/664(0.3%)	6/1,354(0.4%)
2003	0/382(0)	1/259(0.4%)	1/640(0.2%)	2/1,281(0.2%)
2004	7/382(1.8%)	1/252(0.4%)	3/629(0.5%)	11/1,263(0.9%)
2005	11/300(3.7%)	6/257(2.3%)	1/645(0.2%)	18/1,202(1.5%)
2006	15/291(5.2%)	1/243(0.4%)	3/553(0.5%)	19/1,087(1.7%)
2007	13/281(5.5%)	5/235(2.1%)	9/515(1.7%)	27/987(2.7%)
2008	9/268(3.8%)	6/233(2.6%)	10/526(1.9%)	25/997(2.5%)
2009	15/240(5.3%)	8/224(3.6%)	16/530(3.0%)	39/1,035(3.8%)
2010	11/238(4.1%)	10/219(4.6%)	10/416(2.4%)	31/903(3.5%)
2011	21/237(8.8%)	21/238(8.8%)	12/358(3.4%)	54/836(6.4%)
Total	106/3,048(3.5%)	59/2,421(2.4%)	67/5,476(1.2%)	232/10,945(2.1%)
Trend	P<0.001	P<0.001	P<0.001	P<0.001

*Ane*, *Anesthesiology*; *BJA*, *British Journal of Anaesthesia*; *A&A*, *Anesthesia & Analgesia*.

During the study period, the ECA position in the byline varied much. The first two authors with ECAs were used most of the time (*Ane*, 85.8%; *BJA*, 89.8%; *A&A*, 82.1%; totally, 84.9%) (Table 2). The second most common were the last two authors to be credited equally. The median numbers of authors listed in byline of the ECA articles in the three journals were 7 in *Ane*, 6 both in *BJA* and *A&A*. The median number of ECAs in all the three journals was two. The largest numbers of ECAs were 6 in *Ane*, 4 both in *BJA* and *A&A*.

**Table 2:** Number of original research articles with authors given equal credit categorized by byline position.

Byline position of authors receiving equal credit	*Ane* (n = 106)	*BJA* (n = 59)	*A&A* (n = 67)	Total (n = 232)
First two authors	91	53	55	197
First 3 or more authors	2	1	2	5
First two and last two authors	1	1	2	4
First and last authors	0	0	3	3
First two and last authors	1	0	0	1
Middle authors only	2	1	2	5
First 3 (or more) authors and last (or more) authors	2	0	0	2
Last two authors	6	3	3	11
Third and last authors	1	0	0	1

In addition, the articles with ECAs originated from various countries around the world (Table 3), and Western Europe published the largest number of original research articles with ECAs (108), followed by Asia (excluding Japan) (54) and USA (51).

**Table 3:** Regions in original research articles with authors given equal credit, 2002–2011.

Byline position of authors receiving equal credit	*Ane* (n = 106)	*BJA* (n = 59)	*A&A* (n = 67)	Total (n = 232)
USA	31	2	18	51
Western Europe	47	41	20	108
Canada	2	3	3	8
Asia (excluding Japan)	19	12	23	54
Japan	7	1	3	11

Finally, in the instructions to authors of the journals, only *A&A* required authors to clarify their contributions and none of the three journals made any clear reference to stating ECAs in their author form. In the journal *Anaesthesia*, statements such as ‘Author A and author B contributed equally to this paper’ are even forbidden.

## Discussion

This study showed that the practice of giving authors equal credit in original research papers published in major anaesthesia journals increased significantly in the past ten years. The application of giving authors equal credit was found in almost every position in the byline and the most common was the first two authors most of the time. It remained uncommon to have more than two authors with equal contributions. The original research articles with ECAs originated from various regions all over the world. These findings seemed to suggest that it was difficult to accurately discern the contributions of authors based only on their byline positions [7].

Our study showed significant increasing trends in annual proportion of the articles with ECAs in major anaesthesia journals. Although many factors may account for the results, a major aspect is that, currently, more collaborative and multicentered studies are conducted than before. Besides, ECA is a new concept to the researchers, and recently the researchers may gradually accept this concept and apply it when ordering the authors. In evaluating the potential interpretation of equal contribution of authorship for promotion, it is needed to consider the recent policies of the National Institutes of Health (NIH) of USA. In the NIH roadmap, collaborative research teams are clearly emphasized, and dual principal investigators in grant application are often allowed now. These concentrations on close collaboration hint that the number of original articles with multiple authors or senior investigators listed having essentially equal contributions will likely continue to increase.

A manuscript comprises of a great deal of works before its publication, such as study idea, study design, grant application, research collaboration, research practice, data summary, manuscript writing, manuscript revision, and etc. It is reasonable that some authors are given equal credit when they had similar contribution. Currently, many journals required authors to clarify their contributions at the submission of manuscript. However, in the instructions to authors of the four major anaesthesia journals, only *A&A* rigorously required authors to clarify their contributions [8]. This might explain why *A&A* had fewer articles (1.2%) with authors given equal credit than the other two journals. In addition, none of the journals made any clear reference to state ECAs in their author form that it is permitted to have authors credited for having “contributed equally” to a study.

In the study, the yearly proportion of articles with ECAs rose rapidly in 2005–2006, which was also found in another two studies [4], [5]. This might be due to that two foundational bibliometric indices, the Hirsch index [9] and the g-index [10] were founded in 2005–2006. With the appearance of these indices, evaluation committees (such as tenure committees and academic promotion) begin to envision ranking tools that extended beyond bedrock impact factors [11]. Authorship position, impact factor, and citations are easily enrolled into a convenient index. In this condition, it is understandable and not surprising that some investigators would like to spend certain time jockeying in authorship position: the attribution of credit along with their grant application, position promotion, prestige, and income may hinge on it.

It is possible that the yearly proportion of articles with ECAs will continue to rise in the near future, in case that these anaesthesia journals would not standardize the use of equally credited authors. Because the ECA proportion of *A&A* was markedly lower than the other journals, standardization of equally credited authors may reduce the ECA proportion. The standardization of equally credited authors should consist of clarification of their detailed contributions, clear policy to state ECAs and even a formal declaration of ECAs. As another choice, the journals could prohibit the use of ECAs, just like *Anaesthesia*.

The potential limitation of this study was that this study focused on the four major anaesthesia journals and may not be representative of other anaesthesia journals and those from other specialties.

In conclusion, the practice of giving authors equal credit in original research papers is increasingly common in major anaesthesia journals. It may be warranted for the journals to guide the authors how to regard this practice.
